# Evidence from neuroimaging to explore brain plasticity in humans during an ultra-endurance burden

**DOI:** 10.1186/1741-7015-10-171

**Published:** 2012-12-21

**Authors:** Stéphane Perrey, Kevin Mandrick

**Affiliations:** 1 Movement to Health (M2H), Montpellier-1 University, EuroMov, 700 Avenue du Pic Saint Loup - 34090 Montpellier, France; 2 Bodysens, 442 Rue Georges Besse, Immeuble Innovation 3 - 30035 Nîmes, France

**Keywords:** cerebral atrophy, exercise behavior, fatigue, overload, plasticity, running

## Abstract

Physical activity, likely through induction of neuroplasticity, is a promising intervention to promote brain health. In athletes it is clear that training can and does, by physiological adaptations, extend the frontiers of performance capacity. The limits of our endurance capacity lie deeply in the human brain, determined by various personal factors yet to be explored. The human brain, with its vast neural connections and its potential for seemingly endless behaviors, constitutes one of the final frontiers of medicine. In a recent study published in *BMC Medicine*, the TransEurope FootRace Project followed 10 ultra-endurance runners over around 4,500 km across Europe and recorded a large data collection of brain imaging scans. This study indicates that the cerebral atrophy amounting to a reduction of approximately 6% throughout the two months of the race is reversed upon follow-up. While this study will contribute to advances in the limits of human performance on the neurophysiological processes in sports scientists, it will also bring important understanding to clinicians about cerebral atrophy in people who are vulnerable to physical and psychological stress long term.

See related research article http://www.biomedcentral.com/1741-7015/10/170

## Background

A plethora of evidence exists to show that there are no major doubts behind the physical benefits of exercising. Another source of short-term exercise benefit has to do with increased neurogenesis in the brain and improved mental performance [1]. Structural neuroplasticity - the brain's ability to undergo structural alterations following environmental changes - has become an accepted biological phenomenon. However, some questions remain. First, do endurance exercise responses when experienced multiple times over weeks impact the brain in such a way as to become neuroprotective? Second, how do the brain's resources contribute to optimizing running performance? Physiological data on human brain activity during chronic exercise responses are still sparse.

Recently, research on the favorable effects of exercise on the brain structure and function is emerging based on functional neuroimaging approaches (for example, [2]). Repeated and strenuous running exercises constitute a remarkable physical stressor requiring important mental effort. An example of this is the 64 day ultramarathon covering about 4,500 km from Bari, Italy to the North Cape, Norway used in the study of Freund et al. [3], published in *BMC Medicine*. We expect the ultramarathon model to involve concurrent physical and mental demands. Thus, it can probably be considered as one of the main challenges that would allow us to explore the finite resources of the human brain under natural field settings. Freund et al. [3] have successfully explored some biometrical data and the cerebral structural changes occurring in 10 runners in response to chronic endurance running exposure, with the help of three identical magnetic resonance (MR) scanners (one was a mobile MR unit mounted on a truck trailer across Europe). The results of this unique study, which revealed no brain lesions, suggest that ultra-endurance runners likely have important mental effort prerequisites and an adapted training workload to cope with demands of ultramarathon running. Additionally, it gave some clues to the effect of extreme fatigue with energy deficits on changes to the cortical grey matter volume. Having an understanding of what the brain does during an ultramarathon event could help refine research on the matter of mind over muscle [4,5] into the mechanisms determining exercise tolerance. Such a study may benefit not only endurance athletes but also military personnel involved in physical work over prolonged periods and patients affected by unexplained chronic fatigue syndromes.

### Neurophysiological and behavioral changes through extreme running exercise

The study by Freund et al. [3] offers outstanding opportunities that we highlight here. Even with some expected technical difficulties and limitations (sample size), the follow-up at three or four time-points explored the adaptation of different functional body systems, such as changes in body weight and brain volume. In particular, a large data collection of brain imaging scans with dedicated sequence parameters was achieved before, twice during and about eight months after the race (in seven runners). Thus, this study examined whether the grey matter volume changes were driven by the extreme level in physical loading. The observed grey matter atrophy (as an expression of neuronal downregulation), amounting to a reduction of approximately 6% throughout the two months of the race, was reversible on follow-up. A robust literature documents that exercise is a simple and widely practiced behavior that activates molecular and cellular cascades that support and maintain brain health and neuroplasticity [1]. It is typically suggested that acute exercise improves brain functions by increasing cerebral blood flow, and that one source of cognitive benefit is purely cardiovascular [6]. Until now, how the cerebral volume changes with regards to extreme physical load during prolonged exercise has not been documented. Such studies could give some new insights into the prescription of preventative and therapeutic exercise for various diseases, such as Alzheimer's and Parkinson's, that progress via the loss of neurons. Even if cerebral alterations, based on volumetric changes, were observed in the study by Freund et al. [3], although without brain lesions, one may assume that the pattern of ultramarathon exercise-related activation change could produce a functional reorganization of brain network activity. In this reorganization, the pattern of increasing and decreasing activation could occur across distinct brain areas, for example, in the prefrontal cortex, involved daily in motivational processes, and the insula and anterior cingulate cortex, for processing levels of exertion. Extreme physical and psychological stresses, such as those encountered during an ultramarathon task, strongly perturb the body and mind, which in turn initiates complex cognitive and affective response strategies. It remains to be seen whether cognitive changes could influence cerebral structure, independent of the level of physical load.

There is evidence from ecologically valid conditions (that is, non-laboratory settings) that sensory-motor integration tasks involving large-scale bodily movements such as running with specific control and/or attention processes require massive and sustained neural activation of the sensory, motor and autonomic systems [7]. This is coupled with the fact that the brain operates on a fixed amount of metabolic resources [8]. Runners are likely motivated by a set of attention and control areas of the brain that are used to support or cope with novel demands that occur during effortful performance with a changing environment [9]. The mental and physical costs incurred by each runner in the Freund et al. paper [3] while achieving a particular level of performance, taking into account their capacities, should vary - with continuously shifting priorities over time. The more efficient the mental processes are, the better the brain is at utilizing knowledge and experience in decision making, especially under extreme conditions [10]. Among the many methods of measuring mental workload, physiological measures offer promise because they can be more closely linked to brain function. However, more research is needed to clarify how exercise might affect mental ability in the long term by new or complementary research tools. During exercise, mental effort is associated with an increase in cerebral oxygenation [11], which could be locally monitored during exercise by a new emerging neuroimaging technique, the near-infrared spectroscopy (NIRS). Similar to functional MR imaging (fMRI), functional NIRS (fNIRS) is a hemodynamic-based technique for the assessment of functional activity in the human cortex and has the advantage of being relatively resistant to motion artifacts, allowing easy measurement in less restricted and noisy conditions [12]. Emerging portable and miniaturized fNIRS systems open new opportunities to study motor, sensory and autonomic functions in realistic environments in future projects dealing with exercise stress. Future fused fMRI and fNIRS studies have the possibility to delineate potential brain response signatures to study the response of individuals to demanding psychological and physical conditions in an outpatient setting.

## Conclusion and perspectives

From a perspective of neuroimaging, the findings of the study by Freund et al. are significant in that they provide a first view of the dynamic state of brain plasticity as regards long-term running exercise. This project is finding widespread application in the study of chronic human brain activation, motivating further application-specific development of the technology for capturing brain function changes in realistic environments. Study of fatigue in the clinical setting is complicated by its multifactorial etiology, psychological factors and patients' perceptions. The pathophysiology of the physical fatigue experienced by most patients can be viewed from the perspective of exercise physiology. This article is a clear example of a creative way forward to address new fundamental questions on the impact of fatiguing exercise on mental and physical load.

## Competing interests

The authors declare that they have no competing interests.

## Authors' contributions

SP drafted the manuscript and KM made substantial contributions in revising it. SP and KM read and approved the final manuscript.

## Pre-publication history

The pre-publication history for this paper can be accessed here:

http://www.biomedcentral.com/1741-7015/10/171/prepub
